# Supplementary figures and images for: Protein profile changes during priming explants to embryogenic response in Coffea canephora: identification of the RPN12 proteasome subunit involved in the protein degradation
Source: PeerJ. 2024 Nov 11;12:e18372. doi: 10.7717/peerj.18372 (PMC11562780; doi:10.7717/peerj.18372)

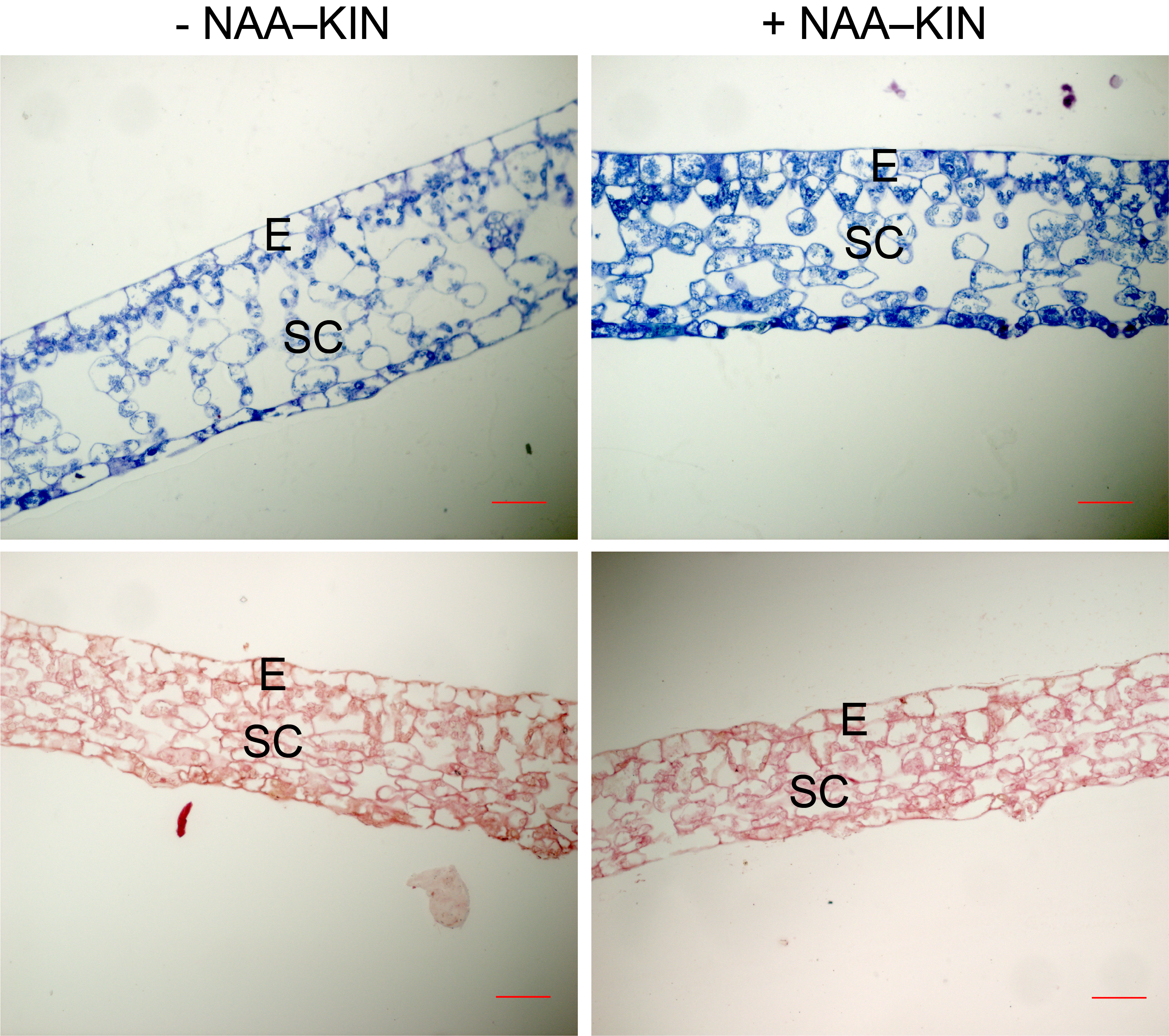

Supplement: Supplemental Information 1 — JB-4 resin sections were stained either with Toluidine blue (upper panel) or Xylidine Ponceau (lower panel). E and SC correspond to the epidermis and spongy mesophyll cells. Bars 20 µm. [file peerj-12-18372-s001.png]

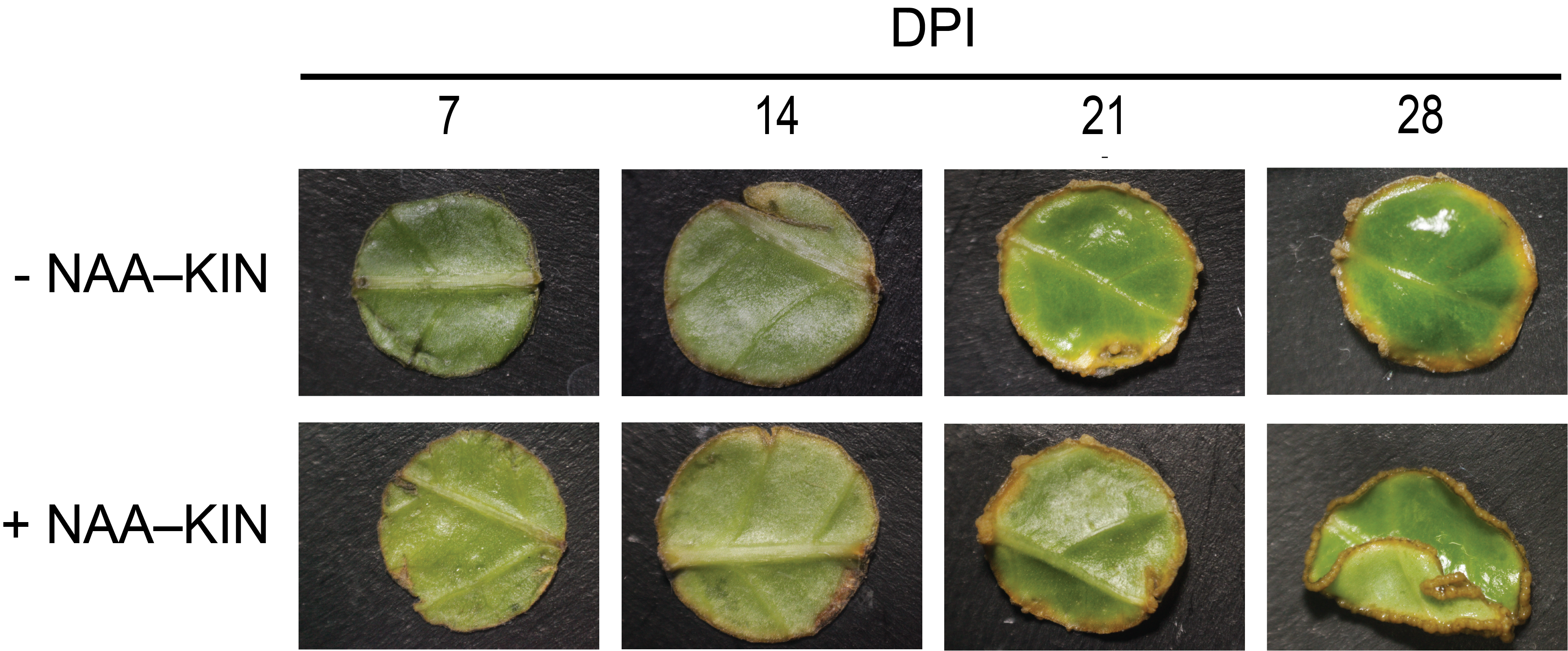

Supplement: Supplemental Information 2 [file peerj-12-18372-s002.png]

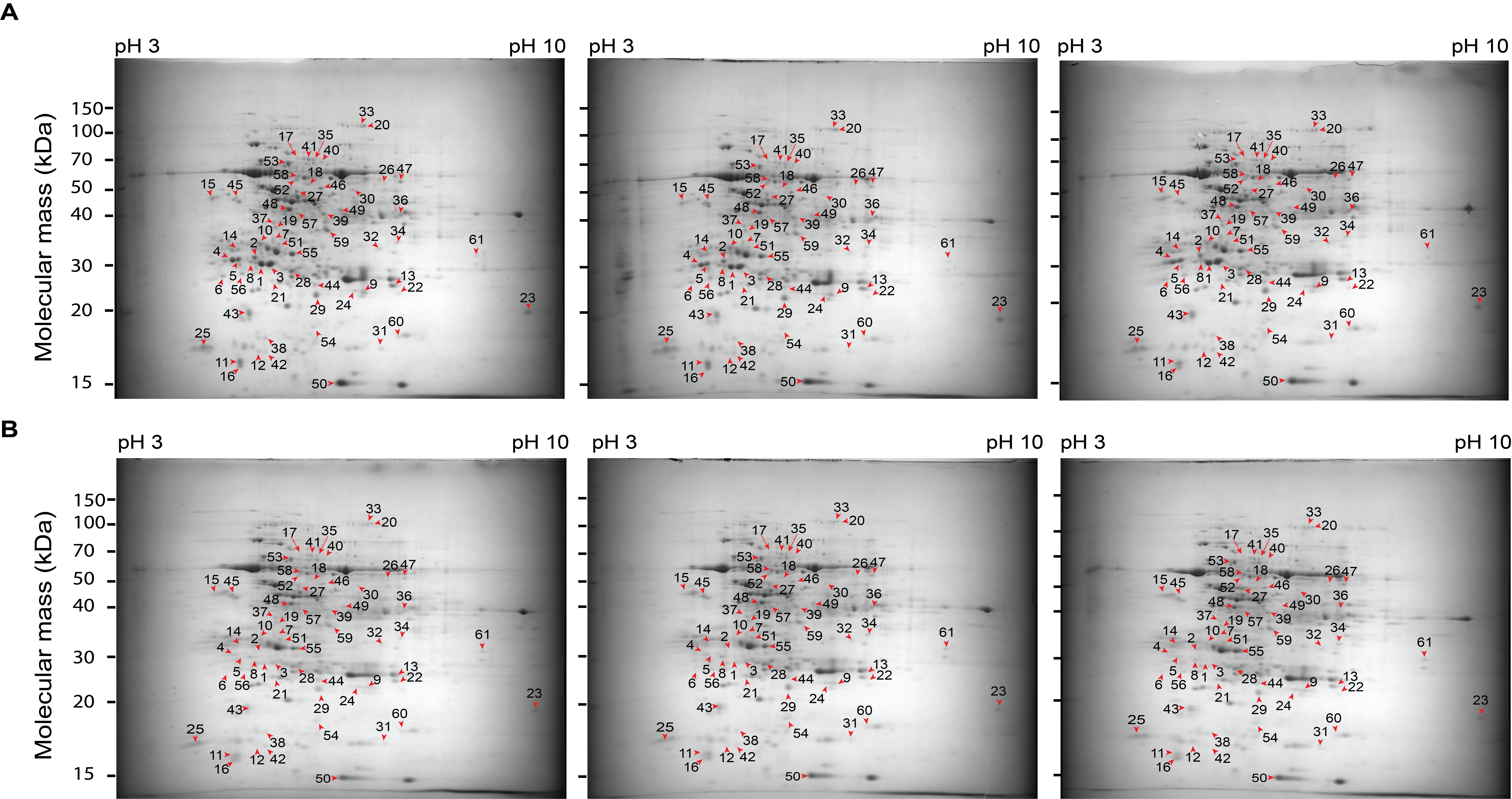

Supplement: Supplemental Information 3 — (A) Representative gel images from cut-leaf + NAA–KIN explants samples. (B) Representative gel images from cut-leaf - NAA–KIN explants samples. Differentially accumulated proteins were stained with colloidal Coomassie G-250. Red arrows indicate differentially accumulated protein spots. [file peerj-12-18372-s003.png]
