# Supplementary material for: Protein profile changes during priming explants to embryogenic response in Coffea canephora: identification of the RPN12 proteasome subunit involved in the protein degradation
Source: PeerJ. 2024 Nov 11;12:e18372. doi: 10.7717/peerj.18372 (PMC11562780; doi:10.7717/peerj.18372)
Supplement: Supplemental Information 4 [file peerj-12-18372-s004.zip › RawCarrillo-Bermejo2Rev/Raw data Primers gels western.docx]

**Auxin and cytokinin cause protein profile alterations to embryogenic response in C*offea canephora***

Evelyn A. Carrillo- ^a^, BermejoLigia Brito-Argáez^b^, Rosa M. Galaz-Ávalos^c^, Víctor M. Loyola-Vargas^d^*, Victor Aguilar-Hernández^e^*

Unidad de Biología Integrativa, Centro de Investigación Científica de Yucatán, Mérida, Yucatán, México.

**Raw data: Primer design for qPCR, primers efficiency calculation, and western blot.**

**> RPN12A _AT1G64520|Arabidopsis thaliana**

ATGGATCCGCAGCTAACTGAAGTTTCACAGCAGTTCGAGAGATTCAAGGCGGCGTTTGCGAGGAAGGATTACAATACCTGTAGCGATCTGTTGTCTCAGCTTAAGGTTCTTCTTACCAAATTCACAAGTCTTCCTCCATTGTTCGAGAATTCACCTAATGCAGCCAAGGAATTGACTATTGCAAGGGACATTTATGAGCATGCTGTTGTTCTAAGCGTCAAAACCGAGGATCAAGATGCTTTTGAGAGAGATTTCTTCCAGCTGAAACCTTACTATGTGGATGCCAGGAACCGTATTCCTCAGTCTCCACAGGAGAATCTTATCCTGGGTCTTAACCTTCTGAGGCTGCTGGTACAAAATAGAATTGCTGAATTCCACACCGAGCTTGAATTACTTTCATCAGCTACACTAGAGGATCCTTGCATCAAGCATGCAGTAGAGCTTGAGCAATCCTTCATGGAGGGTGCCTATAACCGTGTGTTGAGTGCTAGACAGACCGCGCCTGATGCAACTTACGTCTATTTCATGGATCTCTTGGCAAAGACCATAAGAGATGAAATAGCTGGATGCAGCGAGAAAGCTTATGATTATGTCTCTATCAGCGATGCCCGGCAGATGTTGTTGTTCTCTTCTGATCAAGAACTCTTAACCTATGTCACAGATGAGCATCCTGAGTGGGAAGTGAAGGAAGGGTTTGTTGTCTTCCAGAAAGCCAAAGAAACTGCACCGTGCAAAGAGATTCCGTCTCTGCAACTCATCAACCAGACTCTAAGCTACGCCAGAGAGCTGGAGCGTATCGTGTAA

**>RPN12B_AT5G42040|Arabidopsis thaliana**

ATGGATCCGCAACTAATGGAGGTTTCACAGCAGTTCGAGAGGTTTAAAGCTGCGTTTATAATAAAAGATTTCGATACCTGTTCCAGTCTCTTGTCTCAGCTTAAGTTGTTTGATCATTATCTTATATCACTCTCACTGAATGCATTGCTTTTGCTTACGTGTGCTCTGTTTTTTCTATGTACCAGGAATCGTATTCCGCCATCTCCGCAAGAGAATCTAATCATGGGGTTGAACCTACTGAGACTGCTTGTTCAGAACAGAATAGCTGAATTCCACACAGAACTGGGATTACTTTCATCTGCAACCTTGGAGAATCCTTGCATCAAGCATGCGGTGGAGCTCGAGCAATCCTTCATGGAAGGTGCTTATAACCGTGTGTTGAGTGCTAGACAAACCGCACCTGATGAGACTTACGTCTATTTCATGGATCTCTTGGCAAAAACCATTAGAGATGAAATAGCTGGATGCAGTGAGAAAGCATACGATCATCTCTCAATCAGTGAAGGTTGTAAGATGTTACTCTTCTCGTCTGATCAACAACTGTTAACATATGTGAACGAGGAGCACCCGGAGTGGGAAGTTAAAGACGGGTTAGTTGTCTTCCAAAAAACCAGAGAAACCGCACCGTGCAAGGAGATACCGTCACTTCAACTCATCAACCAGACTCTCAGTTACACCAGAGAGCTCGAGCGTATCTTGTAA

**> AT3G18780_ACT2|Arabidopsis thaliana**

ATGGCTGAGGCTGATGATATTCAACCAATCGTGTGTGACAATGGTACCGGTATGGTGAAGGCTGGATTTGCAGGAGATGATGCTCCCAGGGCTGTTTTTCCCAGTGTTGTTGGTAGGCCAAGACATCATGGTGTCATGGTTGGGATGAACCAGAAGGATGCATATGTTGGTGATGAAGCACAATCCAAGAGAGGTATTCTTACCTTGAAGTATCCTATTGAGCATGGTGTTGTTAGCAACTGGGATGATATGGAAAAGATCTGGCATCACACTTTCTACAATGAGCTTCGTATTGCTCCTGAAGAGCACCCTGTTCTTCTTACCGAGGCTCCTCTTAACCCAAAGGCCAACAGAGAGAAGATGACTCAAATCATGTTTGAGACCTTTAACTCTCCCGCTATGTATGTCGCCATCCAAGCTGTTCTCTCCTTGTACGCCAGTGGTCGTACAACCGGTATTGTGCTGGATTCTGGTGATGGTGTGTCTCACACTGTGCCAATCTACGAGGGTTTCTCTCTTCCTCATGCCATCCTCCGTCTTGACCTTGCTGGACGTGACCTTACTGATTACCTCATGAAGATCCTTACAGAGAGAGGTTACATGTTCACCACAACAGCAGAGCGGGAAATTGTAAGAGACATCAAGGAGAAGCTCTCCTTTGTTGCTGTTGACTACGAGCAGGAGATGGAAACCTCAAAGACCAGCTCTTCCATCGAGAAGAACTATGAATTACCCGATGGGCAAGTCATCACGATTGGTGCTGAGAGATTCAGATGCCCAGAAGTCTTGTTCCAGCCCTCGTTTGTGGGAATGGAAGCTGCTGGAATCCACGAGACAACCTATAACTCAATCATGAAGTGTGATGTGGATATCAGGAAGGATCTGTACGGTAACATTGTGCTCAGTGGTGGAACCACTATGTTCTCAGGTATCGCTGACCGTATGAGCAAAGAAATCACAGCACTTGCACCAAGCAGCATGAAGATTAAGGTCGTTGCACCACCTGAAAGGAAGTACAGTGTCTGGATCGGTGGTTCCATTCTTGCTTCCCTCAGCACATTCCAGCAGATGTGGATCTCCAAGGCCGAGTATGATGAGGCAGGTCCAGGAATCGTTCACAGAAAATGTTTCTAA


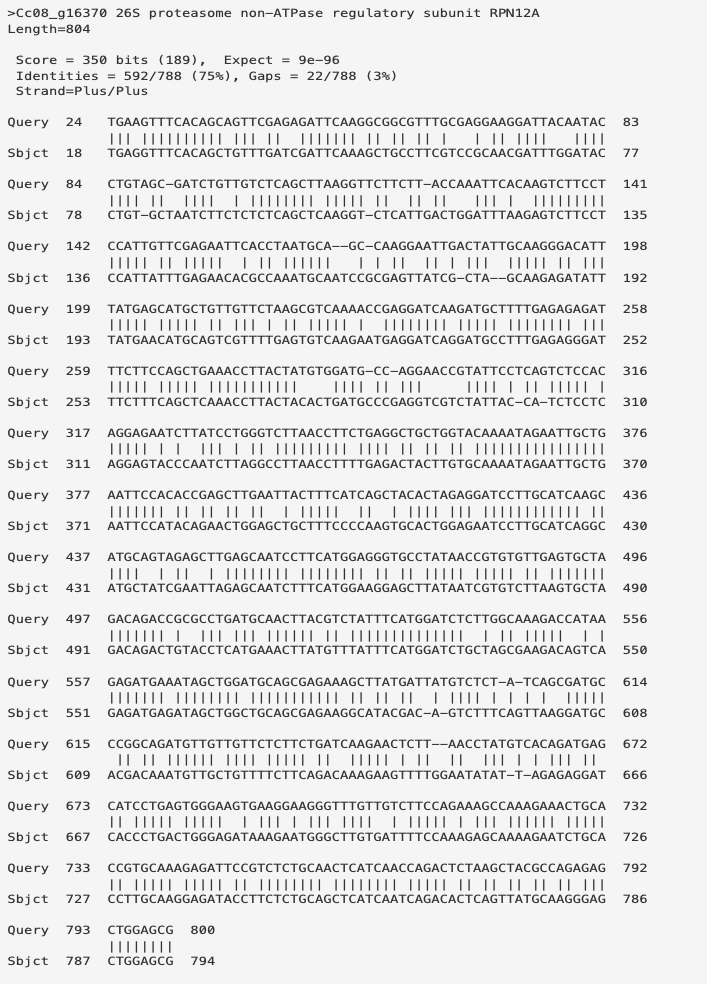


**Blast pairwise alignment of *A. thaliana* *RPN12A* gene query againt *C. canephora* *RPN12***


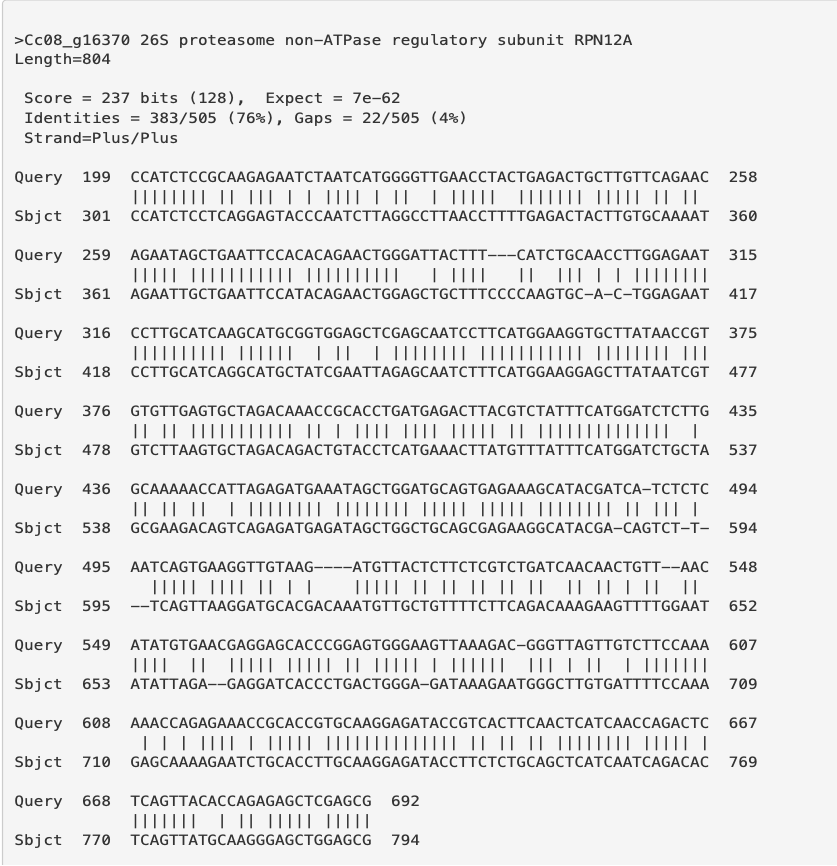


**Blast pairwise alignment of *A. thaliana* *RPN12B* gene query againt *C. canephora* *RPN12***


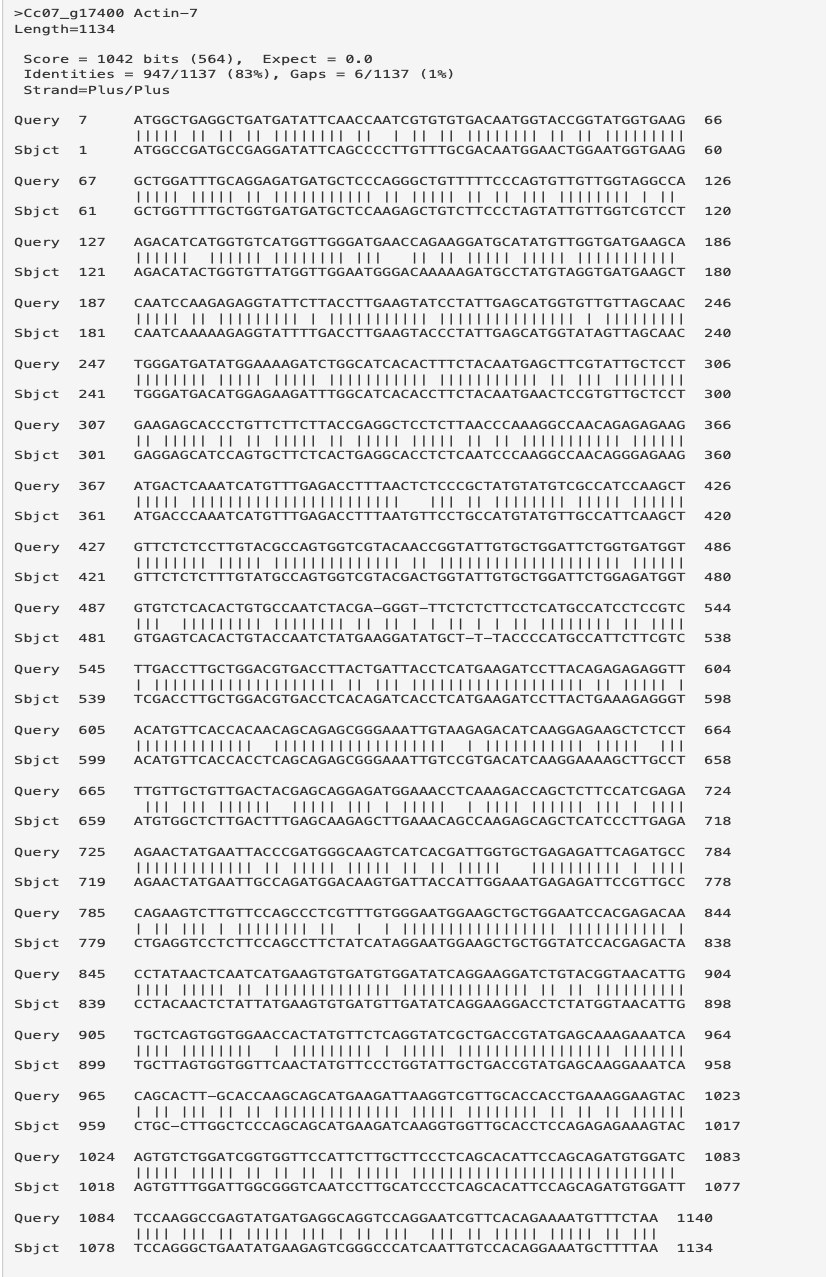


**Blast pairwise alignment of *A. thaliana* *ACT2* gene query againt *C. canephora* *ACT2***

**>Cc08_g16370:1-804 26S proteasome non-ATPase regulatory subunit RPN12|chr8:30903596..30906279| CDS chr8:30902634..30906283|Coffea canephora**

ATGGATCCTAAATTTATTGAGGTTTCACAGCTGTTTGATCGATTCAAAGCTGCCTTCGTCCGCAACGATTTGGATACCTGTGCTAATCTTCTCTCTCAGCTCAAGGTCTCATTGACTGGATTTAAGAGTCTTCCTCCATTATTTGAGAACACGCCAAATGCAATCCGCGAGTTATCGCTAGCAAGAGATATTTATGAACATGCAGTCGTTTTGAGTGTCAAGAATGAGGATCAGGATGCCTTTGAGAGGGATTTCTTTCAGCTCAAACCTTACTACACTGATGCCCGAGGTCGTCTATTACCATCTCCTCAGGAGTACCCAATCTTAGGCCTTAACCTTTTGAGACTACTTGTGCAAAATAGAATTGCTGAATTCCATACAGAACTGGAGCTGCTTTCCCCAAGTGCACTGGAGAATCCTTGCATCAGGCATGCTATCGAATTAGAGCAATCTTTCATGGAAGGAGCTTATAATCGTGTCTTAAGTGCTAGACAGACTGTACCTCATGAAACTTATGTTTATTTCATGGATCTGCTAGCGAAGACAGTCAGAGATGAGATAGCTGGCTGCAGCGAGAAGGCATACGACAGTCTTTCAGTTAAGGATGCACGACAAATGTTGCTGTTTTCTTCAGACAAAGAAGTTTTGGAATATATTAGAGAGGATCACCCTGACTGGGAGATAAAGAATGGGCTTGTGATTTTCCAAAGAGCAAAAGAATCTGCACCTTGCAAGGAGATACCTTCTCTGCAGCTCATCAATCAGACACTCAGTTATGCAAGGGAGCTGGAGCGGATTGTTTGA

| **Programa** | **Forward Primer 5’-3’** | **Tm** | **GC%** | **Reverse Primer 5´-3’** | **Tm** | **GC%** | **Product Length** |
| --- | --- | --- | --- | --- | --- | --- | --- |
| *Primer3 | ATTCAAAGCTGCCTTCGTCC | 58.83 | 50 | CCTCGGGCATCAGTGTAGTA | 58.60 | 55 | 249 |

*** Amplicon is highlighted in yellow.**

**> Actin-7 (ACTIN 2 in At)| GSCOCT00037436001|ID=GSCOCT00037436001| Name=Cc07t17400.1| strand = plus, start = 235, end = 467, size = 233 bp | chr7:14504445..14507436|Coffea canephora**
ATGGCCGATGCCGAGGATATTCAGCCCCTTGTTTGCGACAATGGAACTGG
AATGGTGAAGGCTGGTTTTGCTGGTGATGATGCTCCAAGAGCTGTCTTCC
CTAGTATTGTTGGTCGTCCTAGACATACTGGTGTTATGGTTGGAATGGGA
CAAAAAGATGCCTATGTAGGTGATGAAGCTCAATCAAAAAGAGGTATTTT
GACCTTGAAGTACCCTATTGAGCATGGTATAGTTAGCAACTGGGATGACA
TGGAGAAGATTTGGCATCACACCTTCTACAATGAACTCCGTGTTGCTCCT
GAGGAGCATCCAGTGCTTCTCACTGAGGCACCTCTCAATCCCAAGGCCAA
CAGGGAGAAGATGACCCAAATCATGTTTGAGACCTTTAATGTTCCTGCCA
TGTATGTTGCCATTCAAGCTGTTCTCTCTTTGTATGCCAGTGGTCGTACG
ACTGGTATTGTGCTGGATTCTGGAGATGGTGTGAGTCACACTGTACCAAT
CTATGAAGGATATGCTTTACCCCATGCCATTCTTCGTCTCGACCTTGCTG
GACGTGACCTCACAGATCACCTCATGAAGATCCTTACTGAAAGAGGGTAC
ATGTTCACCACCTCAGCAGAGCGGGAAATTGTCCGTGACATCAAGGAAAA
GCTTGCCTATGTGGCTCTTGACTTTGAGCAAGAGCTTGAAACAGCCAAGA
GCAGCTCATCCCTTGAGAAGAACTATGAATTGCCAGATGGACAAGTGATT
ACCATTGGAAATGAGAGATTCCGTTGCCCTGAGGTCCTCTTCCAGCCTTC
TATCATAGGAATGGAAGCTGCTGGTATCCACGAGACTACCTACAACTCTA
TTATGAAGTGTGATGTTGATATCAGGAAGGACCTCTATGGTAACATTGTG
CTTAGTGGTGGTTCAACTATGTTCCCTGGTATTGCTGACCGTATGAGCAA
GGAAATCACTGCCTTGGCTCCCAGCAGCATGAAGATCAAGGTGGTTGCAC
CTCCAGAGAGAAAGTACAGTGTTTGGATTGGCGGGTCAATCCTTGCATCC
CTCAGCACATTCCAGCAGATGTGGATTTCCAGGGCTGAATATGAAGAGTC
GGGCCCATCAATTGTCCACAGGAAATGCTTTTAA

| **Programa** | **Forward Primer 5’-3’** | **Tm** | **GC%** | **Reverse Primer 5´-3’** | **Tm** | **GC%** | **Product Length** |
| --- | --- | --- | --- | --- | --- | --- | --- |
| *Primer 3 | AGCAACTGGGATGACATGGA | 59.01 | 50 | TCCAGCACAATACCAGTCGT | 59.03 | 50 | 233 |

*** Amplicon is highlighted in yellow.**

**Table 1. Nucleic acid quantification, yield, and purity.**

| **Samples** | **Concentration ng/μL** | **260/280 OD ratio** | **260/230 OD ratio** |
| --- | --- | --- | --- |
| T1* | 261.4 | 1.91 | 1.77 |
| T1.1 | 240.4 | 1.99 | 1.85 |
| T1.3 | 293.8 | 1.9 | 1.7 |
| T2* | 335.1 | 1.89 | 1.89 |
| T2.1 | 353.4 | 1.91 | 1.79 |
| T2.3 | 315.4 | 1.93 | 1.87 |

* T1s = explant -AUX -KIN; T2s= explant +AUX +KIN

T1 T1.1 T1.3 T2 T2.1 T2.3


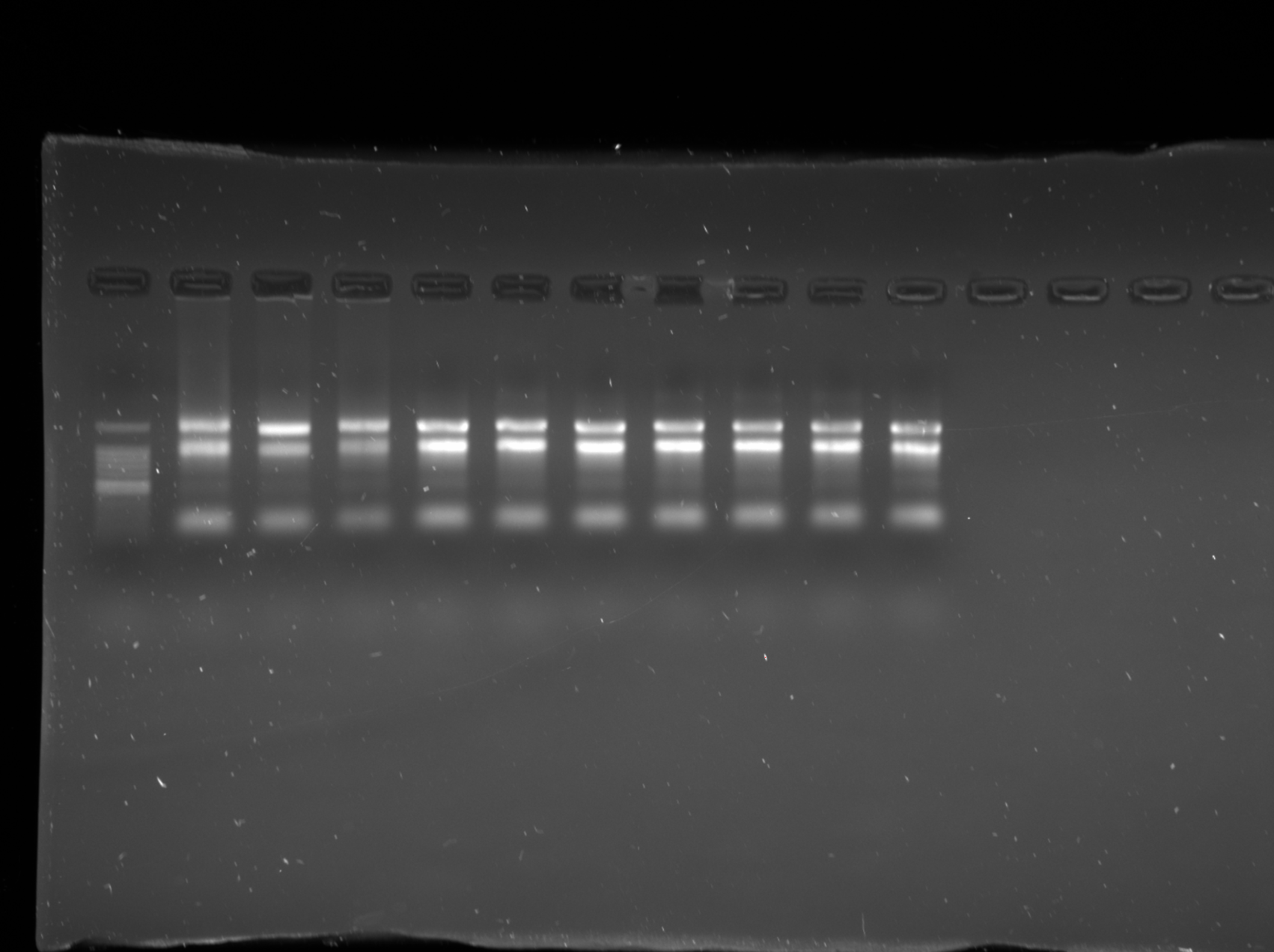


**Agarose gel (1.2%) electrophoresis of RNA samples from plantlets.**

|  |  |  |  |  |  |
| --- | --- | --- | --- | --- | --- |
| **Tube** | **Ct 1** | **Ct 2** | **Avg Ct** | **Sample Quantity** | **Log (sample quantity)** |
| A | 16.11 | 15.92 | 16.015 | 1.0000 | 0.00 |
| B | 19.81 | 19.13 | 19.47 | 0.1000 | -1.00 |
| C | 23.29 | 23.35 | 23.32 | 0.0100 | -2.00 |
| D | 26.39 | 26.54 | 26.465 | 0.0010 | -3.00 |
| E | 25.88 | 25.31 | 25.595 | 0.0001 | -4.00 |
|  |  |  |  |  |  |
|  |  |  |  | **Dilution Factor** | 10 |
|  |  |  |  |  |  |
|  |  |  |  | **Slope** | -2.6155 |
|  |  |  |  | **R Squared** | 0.8922 |
|  |  |  |  | **Efficiency (%)** | **141.18** |
|  |  |  |  |  |  |

## Validating the ACT2 primer and calculating the reaction efficiency. The first point of the standard curve was a cDNA sample. From there, a series of dilutions were performed, 1:10 and 1:100. In this work, three points were used for the standard curve (1, 1:10, 1:100, 1:1000, 1:10000). The Ct values of the technical replicates were averaged. Two technical replicates were used. Then, the logarithmic value of the average of the Cts was determined. The regression slope was obtained from the logarithmic values and the average Ct values. Finally, the efficiency of the primers was calculated as a percentage with the following equation.: Efficiency (%) =$\left( \frac{\boldsymbol{-}\boldsymbol{1}}{\boldsymbol{10}^{\boldsymbol{slope}}\boldsymbol{-}\boldsymbol{1}} \right)\boldsymbol{x} \boldsymbol{100}$.

| **Tube** | **Ct 1** | **Ct 2** | **Avg Ct** | **Sample Quantity** | **Log (sample quantity)** |
| --- | --- | --- | --- | --- | --- |
| A | 17.83 | 18.19 | 18.01 | 1.0000 | 0.00 |
| B | 21.57 | 21.96 | 21.765 | 0.1000 | -1.00 |
| C | 26.11 | 26.56 | 26.335 | 0.0100 | -2.00 |
| D | 28.95 | 30.95 | 29.95 | 0.0010 | -3.00 |
| E | 34.3 | 30.71 | 32.505 | 0.0001 | -4.00 |
|  |  |  |  |  |  |
|  |  |  |  | **Dilution Factor** | 10 |
|  |  |  |  |  |  |
|  |  |  |  | **Slope** | -3.7175 |
|  |  |  |  | **R Squared** | 0.9914 |
|  |  |  |  | **Efficiency (%)** | **85.78** |

## Figure. Validating the RPN12 primer and calculating the reaction efficiency. The first point of the standard curve was a cDNA sample. From there, a series of dilutions were performed, 1:10 and 1:100. In this work, three points were used for the standard curve (1, 1:10, 1:100, 1:1000, 1:10000). The Ct values of the technical replicates were averaged. Two technical replicates were used. Then, the logarithmic value of the average of the Cts was determined. The regression slope was obtained from the logarithmic values and the average Ct values. Finally, the efficiency of the primers was calculated as a percentage with the following equation.: Efficiency (%) =$\left( \frac{\boldsymbol{-}\boldsymbol{1}}{\boldsymbol{10}^{\boldsymbol{slope}}\boldsymbol{-}\boldsymbol{1}} \right)\boldsymbol{x} \boldsymbol{100}$.

+ NAA–KIN


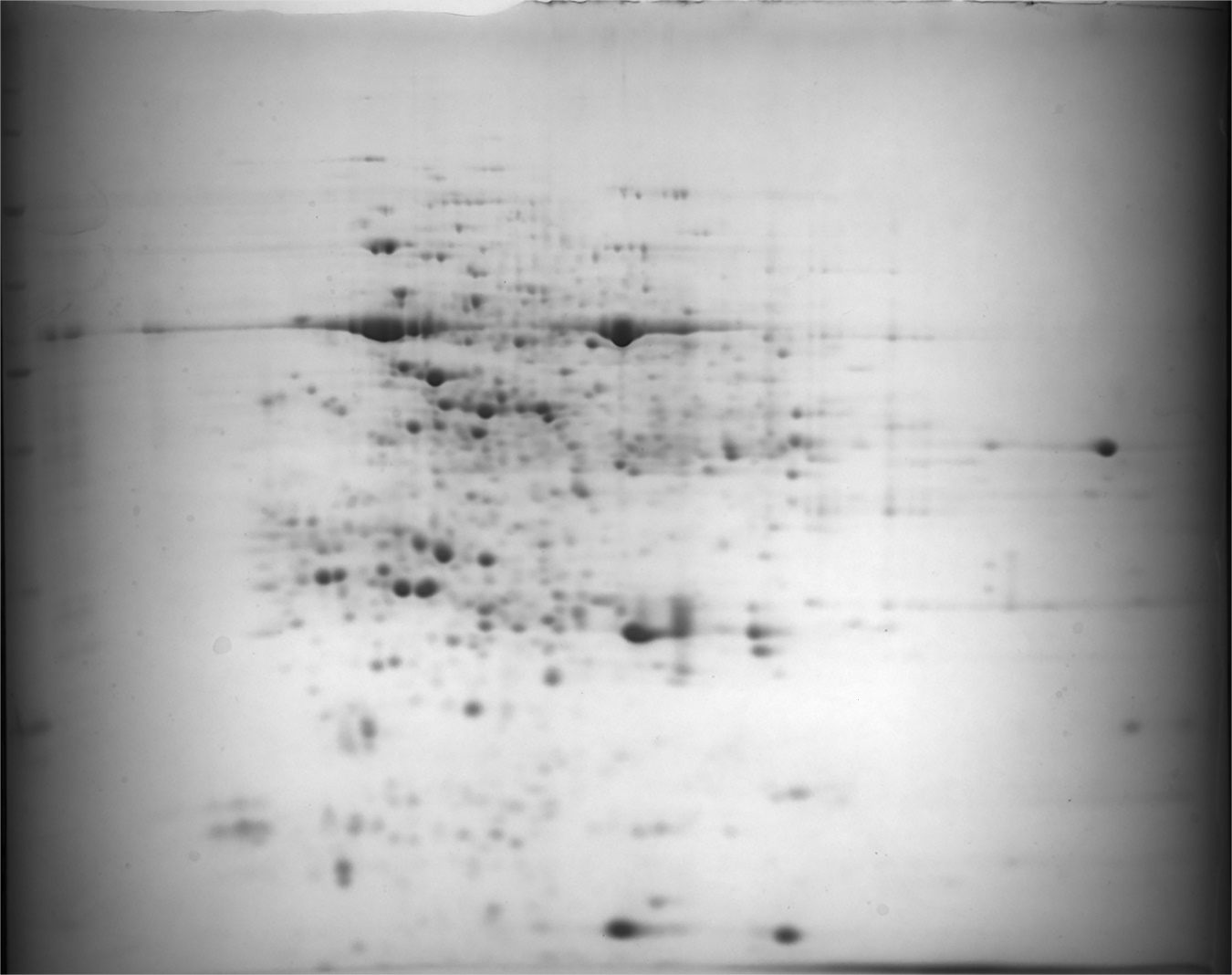


+ NAA–KIN


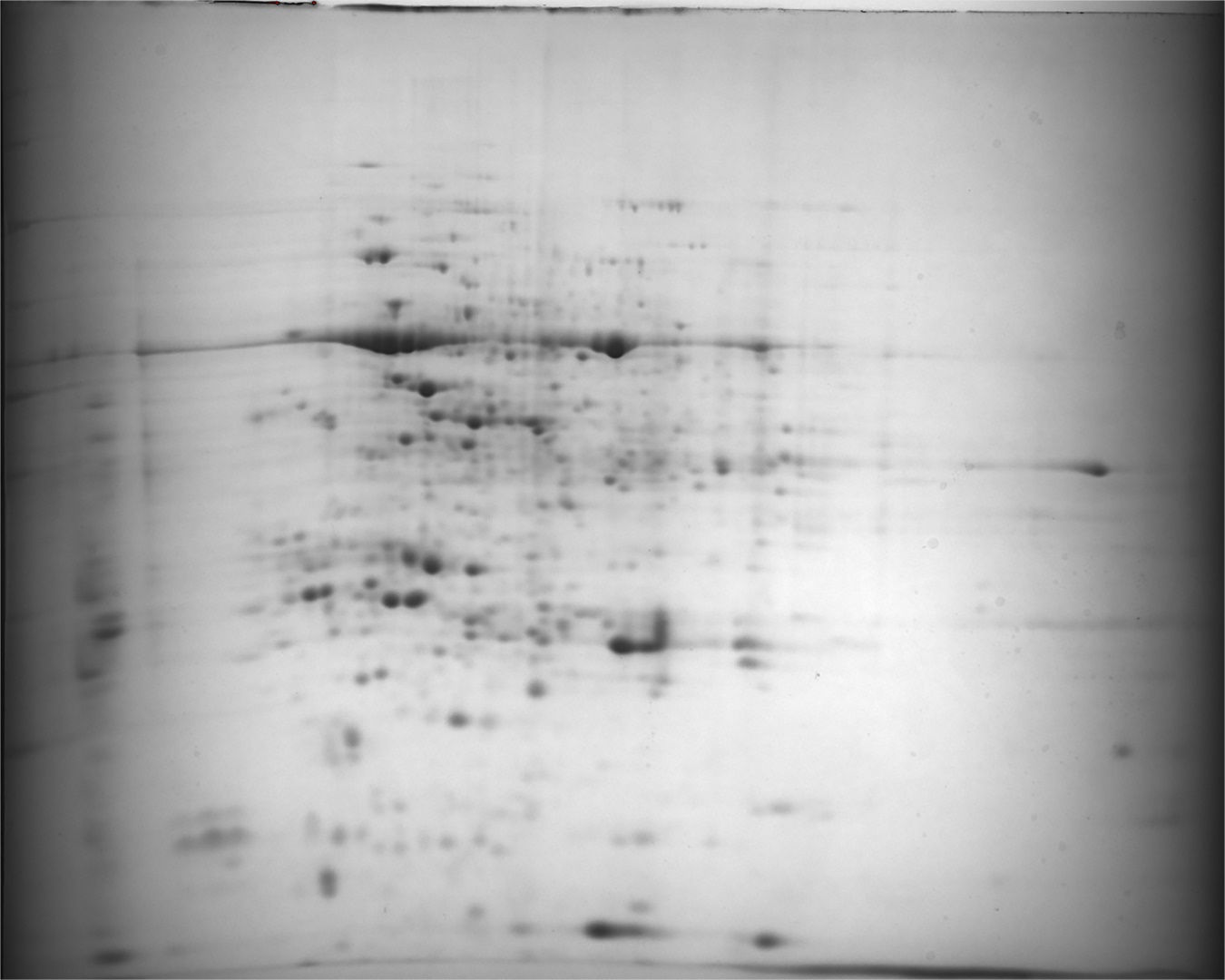


+ NAA–KIN


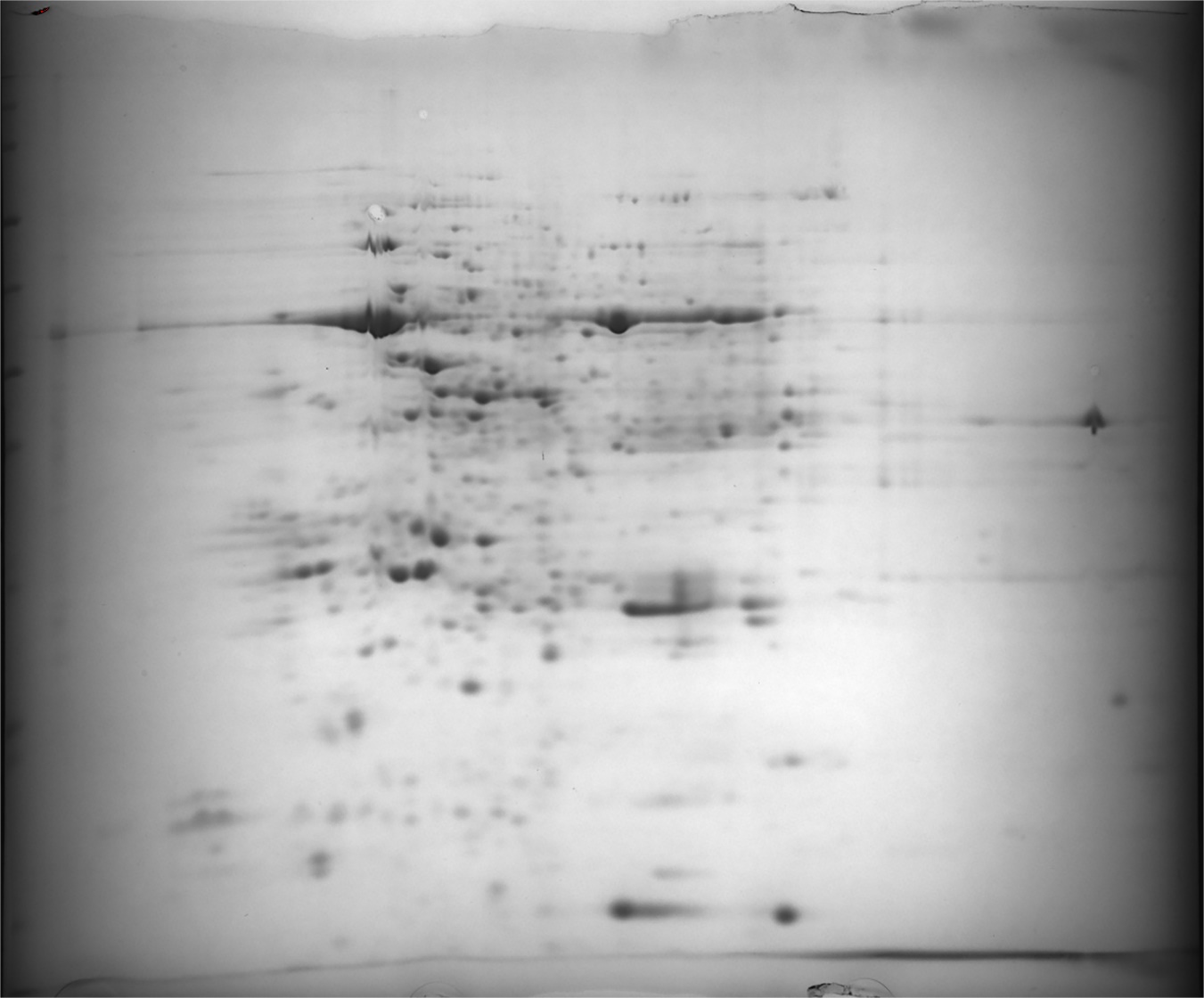


- NAA–KIN


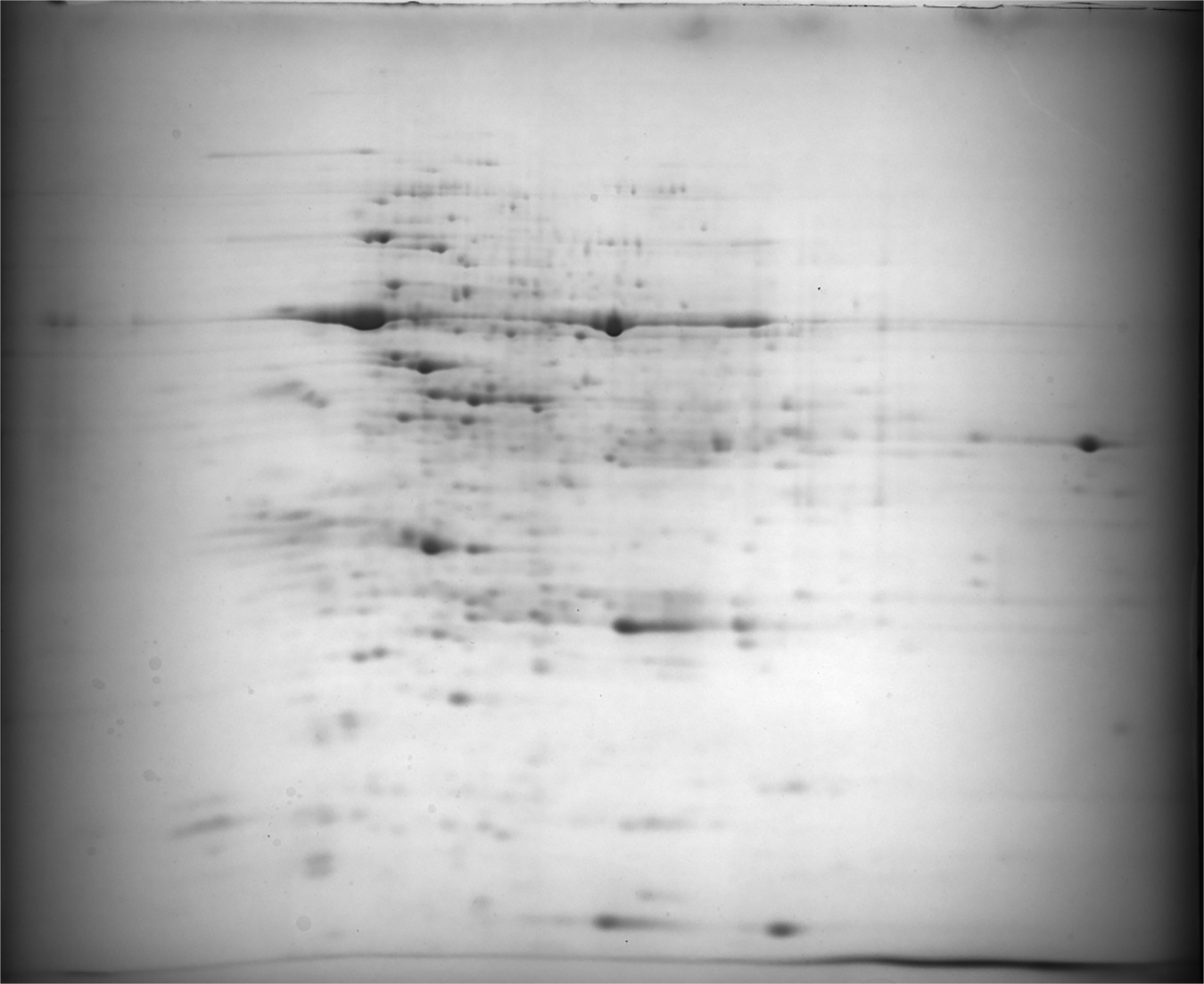


- NAA–KIN


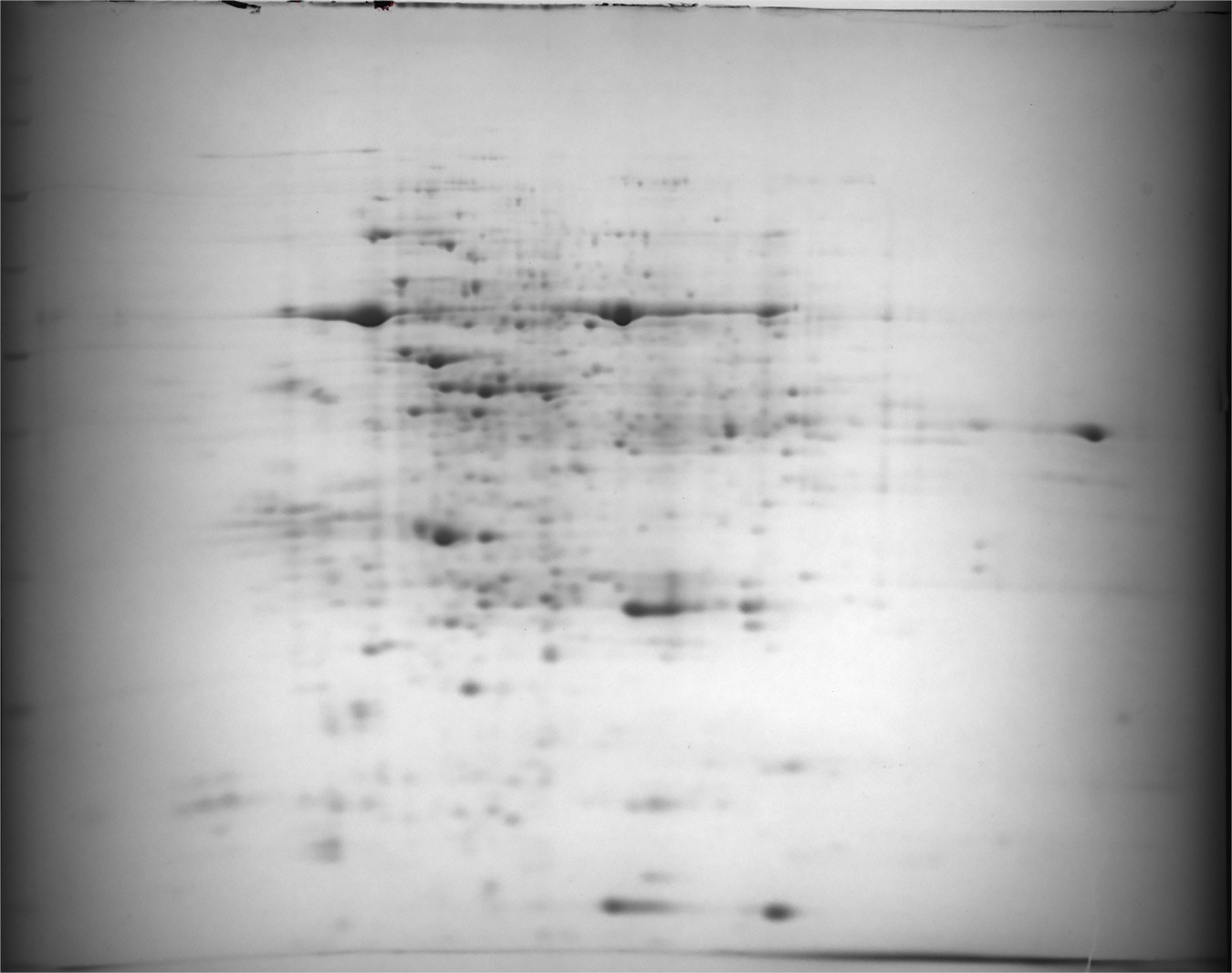


- NAA–KIN


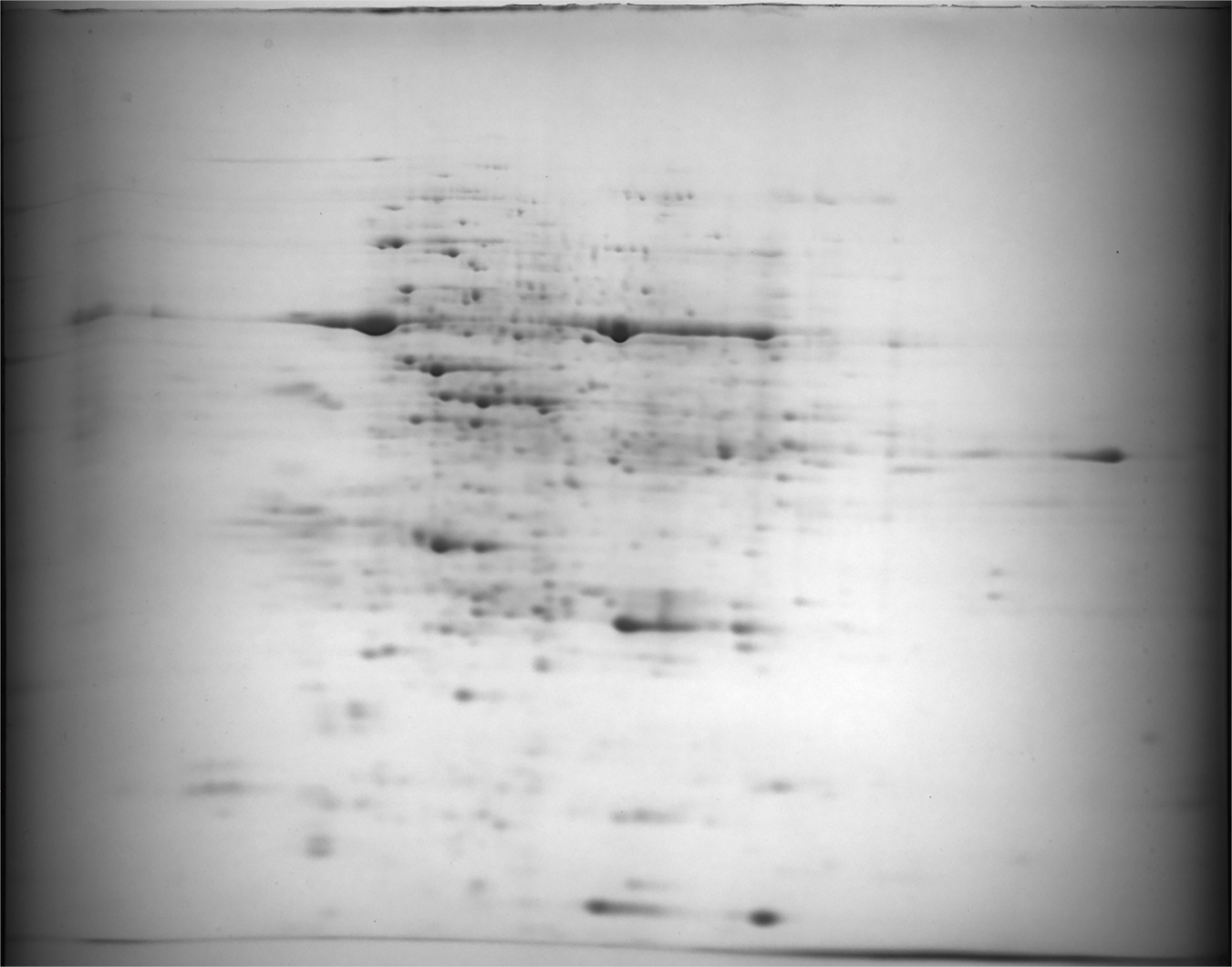


**Original two-dimensional gel electrophoresis images that correspond to Figure 2 and Figure S3.**


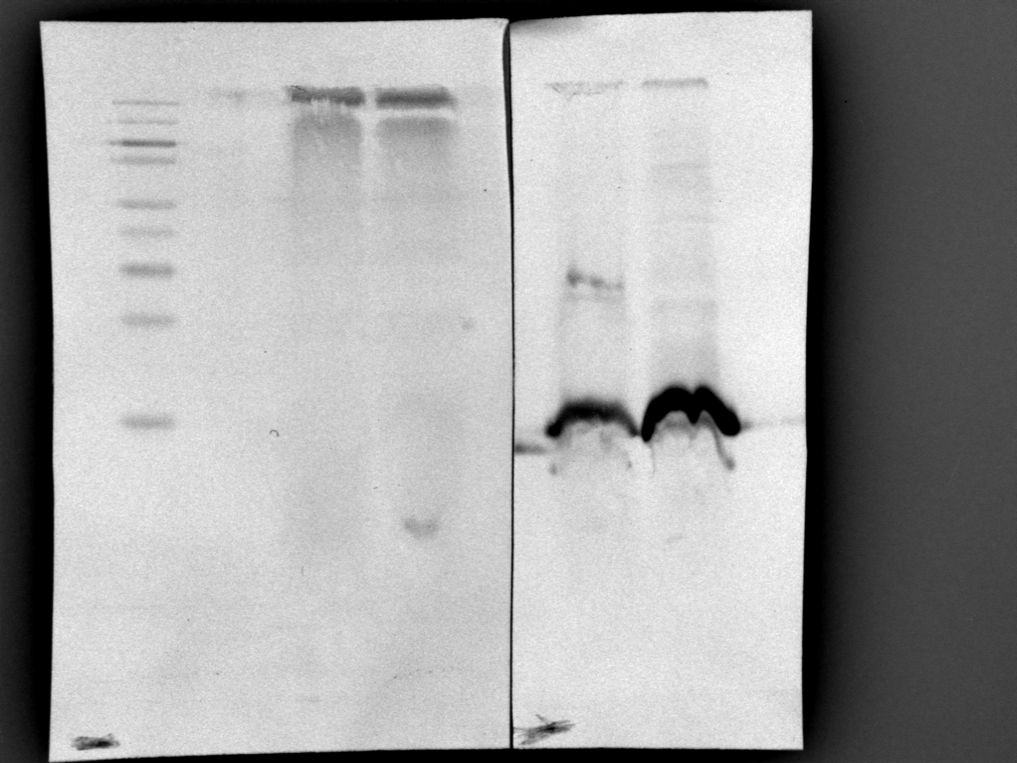


**M**

**Anti-H3**

**Anti-Ub**

**T2**

**T1**

**T2**

**T1**

**25**

**35**

**40**

**50**

**kDa**

**15**

**70**

**100**

**140**

**260**

**Original western blot image that corresponds to Figure 3A.** T1 corresponds to - NAA–KIN and T2 corresponds to + NAA–KIN. M, spectra multicolor broad range protein ladder (Thermo Scientific).
