# Supplementary material for: Protein profile changes during priming explants to embryogenic response in Coffea canephora: identification of the RPN12 proteasome subunit involved in the protein degradation
Source: PeerJ. 2024 Nov 11;12:e18372. doi: 10.7717/peerj.18372 (PMC11562780; doi:10.7717/peerj.18372)
Supplement: Supplemental Information 5 [file peerj-12-18372-s005.docx]

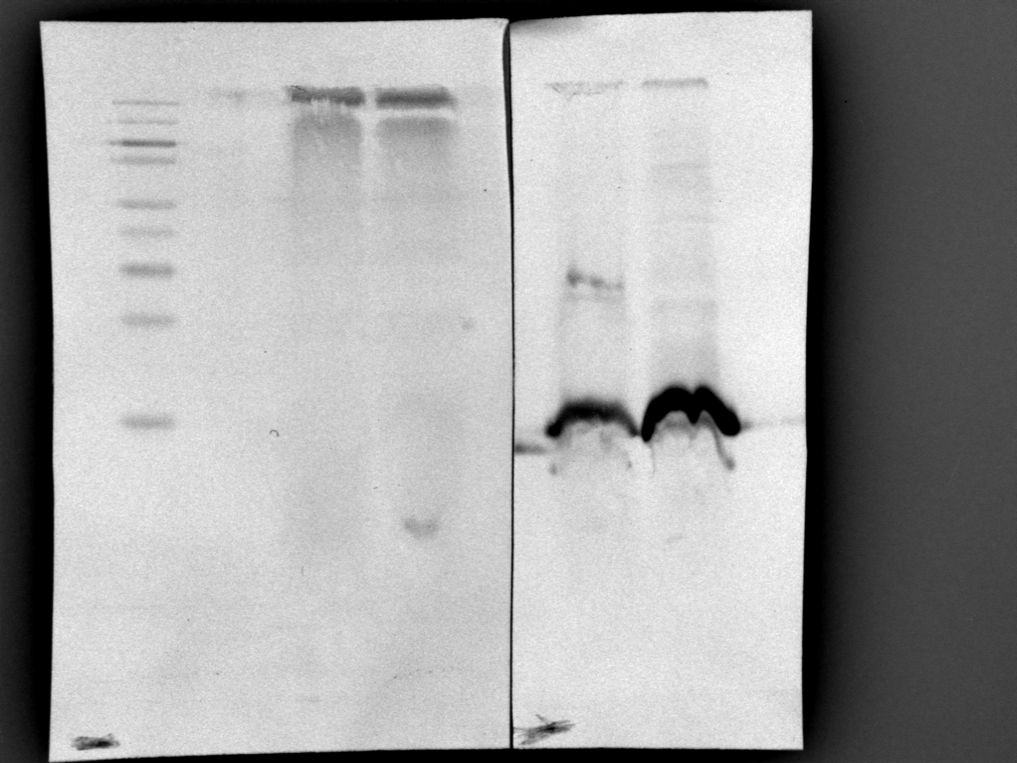


**M**

**Anti-H3**

**Anti-Ub**

**T2**

**T1**

**T2**

**T1**

**25**

**35**

**40**

**50**

**kDa**

**15**

**70**

**100**

**140**

**260**

**Original western blot image that corresponds to Figure 3.** T1 corresponds to - NAA–KIN and T2 corresponds to + NAA–KIN. M, spectra multicolor broad range protein ladder (Thermo Scientific).
